# Who will benefit more from maintenance therapy of metastatic colorectal cancer?

**DOI:** 10.18632/oncotarget.23549

**Published:** 2017-12-21

**Authors:** Mingyi Zhou, Lingyu Fu, Jingdong Zhang

**Affiliations:** ^1^ Department of Gynecology, Cancer Hospital of China Medical University, Liaoning Cancer Hospital and Institute, Shenyang 110042, Liaoning Province, PR China; ^2^ Department of Clinical Epidemiology and Evidence Based Medicine, The First Hospital of China Medical University, Shenyang 110001, Liaoning Province, PR China; ^3^ Department of Medical Oncology, Cancer Hospital of China Medical University, Liaoning Cancer Hospital and Institute, Shenyang 110042, Liaoning Province, PR China

**Keywords:** metastatic colorectal cancer, maintenance treatment, complete or partial response, stable disease, network meta-analysis

## Abstract

Whether there is a difference in the efficacy of maintenance treatment for metastatic colorectal cancer (mCRC) between patients who achieve complete response (CR)/partial response (PR) and those with stable disease (SD) after induction treatment is controversial. PubMed, Cochrane Systematic Reviews, the Cochrane Collaboration Central Register of Controlled Clinical Trials, ClinicalTrials.gov, and databases of conferences were queried to identify randomized controlled trials evaluating the efficacy of maintenance treatment for mCRC patients. The search included articles dated from the inception of these resources until June 20, 2017. We estimated hazard ratios (HRs) for progression-free survival (PFS) and overall survival (OS). Network meta-analysis was performed to compare the efficacy of four regimens as maintenance treatment. Three randomized controlled trials comprising 1,301 patients were included in this network meta-analysis. Patients who achieved CR/PR after induction therapy benefited more from maintenance treatment than patients who achieved SD (PFS: HR [CR/PR] 1.50, 95% CI 1.09–2.08, vs. HR [SD] 1.35, 95% CI 1.04–1.74; OS: HR [CR/PR] 1.04, 95% CI 0.94–1.15, vs. HR [SD] 1.03, 95% CI 0.99–1.07). The results of network meta-analysis suggested that chemotherapy alone and observation were inferior to chemotherapy plus bevacizumab as maintenance treatment. Patients with mCRC who achieve CR/PR after induction therapy might benefit more from maintenance treatment than those with SD. Chemotherapy plus bevacizumab was the most appropriate regimen for maintenance treatment.

## INTRODUCTION

Metastatic colorectal cancer (mCRC) is a common cancer worldwide. There were an estimated 135,430 new cases and 50,260 deaths in the United States in 2017 [1] and 376,300 new cases and 191,000 deaths in China in 2015 [2]. The treatment of mCRC is complex and the balance between efficacy and toxicities of various regimens should be considered. The results of published papers suggested that maintenance treatment prolonged the progression-free survival (PFS) of mCRC patients, but did not prolong the overall survival (OS). However, there is heterogeneity in recent analyses of the efficacy of maintenance treatment. Patients with complete response (CR)/partial response (PR) and those with stable disease (SD) after induction therapy were enrolled. In addition, the criterion of SD is a less than 20% increase or a less than 30% decrease in the sum of diameters of lesions after induction treatment [3], and some patients with SD might be resistant to induction chemotherapy. There is currently controversy over whether there is a difference in the efficacy of maintenance treatment between patients who achieve CR/PR and those with SD after induction treatment.

## RESULTS

**Figure 1:**
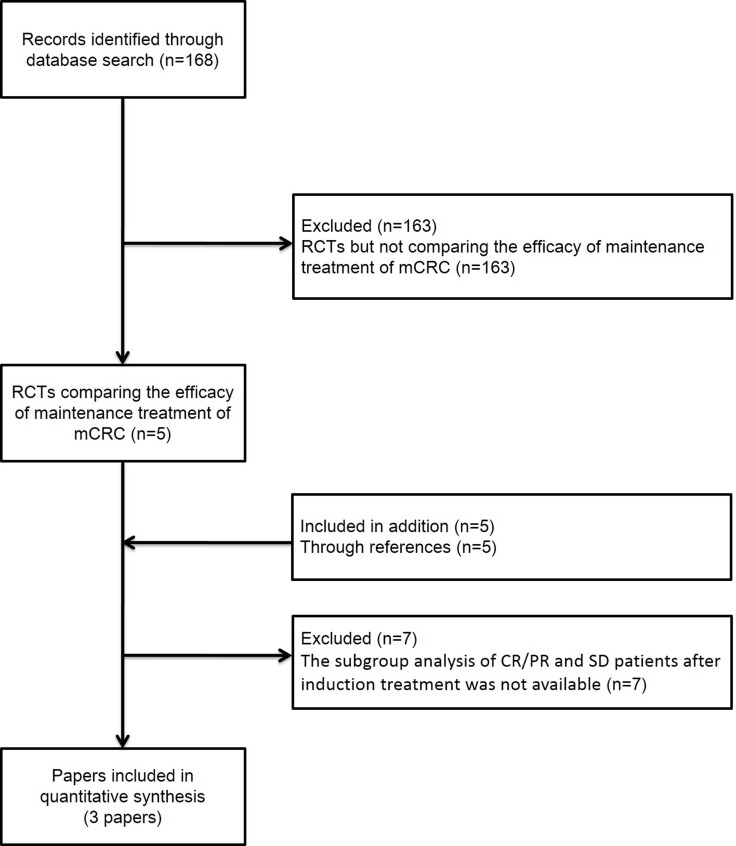
Literature search and selection of studies Abbreviation: RCT, randomized controlled trial.

The titles and abstracts of 168 studies were reviewed. After the initial screen, we assessed potentially eligible papers and selected three papers for further analysis [4–6] (Figure 1). SAKK 41/06, MACRO, XelQuali, OPTIMOX1, OPTIMOX2, OPTIMOX3, and Nordic ACT2 studies were excluded because the necessary data were not available [7–13]. Patients with mCRC (*n* = 1,301) were randomly assigned into maintenance treatment groups versus observation groups (Table 1). Methodological quality assessment was performed according to the latest guidelines in the Cochrane Handbook for Systematic Reviews of Interventions. The quality of each included study was high (Table 2).

**Table 1:** Characteristics of the included randomized control trials

Study	year	Study design	Treatment schedule
CAIRO3	2015	Capecitabine+Bev.	Capecitabine: 625 mg/m^2^ orally twice daily continuously; Bev: 7·5 mg/kg intravenously every 3 weeks
		Observation	
AIO 0207	2015	Fluoropyrimidine+Bev.	Capecitabine 1000 mg/m^2^ orally twice daily on days 1–14, 22–35; or 5-FU 400 mg/m^2^ intravenously bolus, and 5-FU 2400 mg/m^2^ intravenously on days 1, 15, 29; or 5-FU 400 mg/m^2^ intravenously bolus, and 5-FU 600 mg/m^2^ intravenously on days 1, 2, 15, 16, 29, 30; or 5-FU 3000 mg/m^2^ intravenously on days 1, 15, 29 Bev: 7.5 mg/kg every 3 weeks, or 5 mg/kg every 2 weeks.
		Bev	Bev: 7.5 mg/kg every 3 weeks, or 5 mg/kg every 2 weeks.
		Observation	
NCT02027363	2016	Capecitabine	Capecitabine: 1000 mg/m^2^ twice a day from days 1–14, every 3 weeks.
		Observation	

Abbreviations: Bev, Bevacizumab; IV, intravenous.

**Table 2:** Methodological quality of included RCTs

Study	year	Sequence generation	Allocation sequence concealment	Blinding of participants and personnel	Blinding of outcome assessment	Incomplete outcome data	Selective outcome reporting	Other risk of bias
CAIRO3	2015	adequate	adequate	Not report	yes	no	no	no
AIO 0207	2015	adequate	adequate	Not report	yes	no	no	no
NCT02027363	2016	adequate	adequate	Not report	yes	no	no	no

Abbreviation: ITT, intention to treat.

Unclear reporting of allocation was considered inadequate.

**Figure 2:**
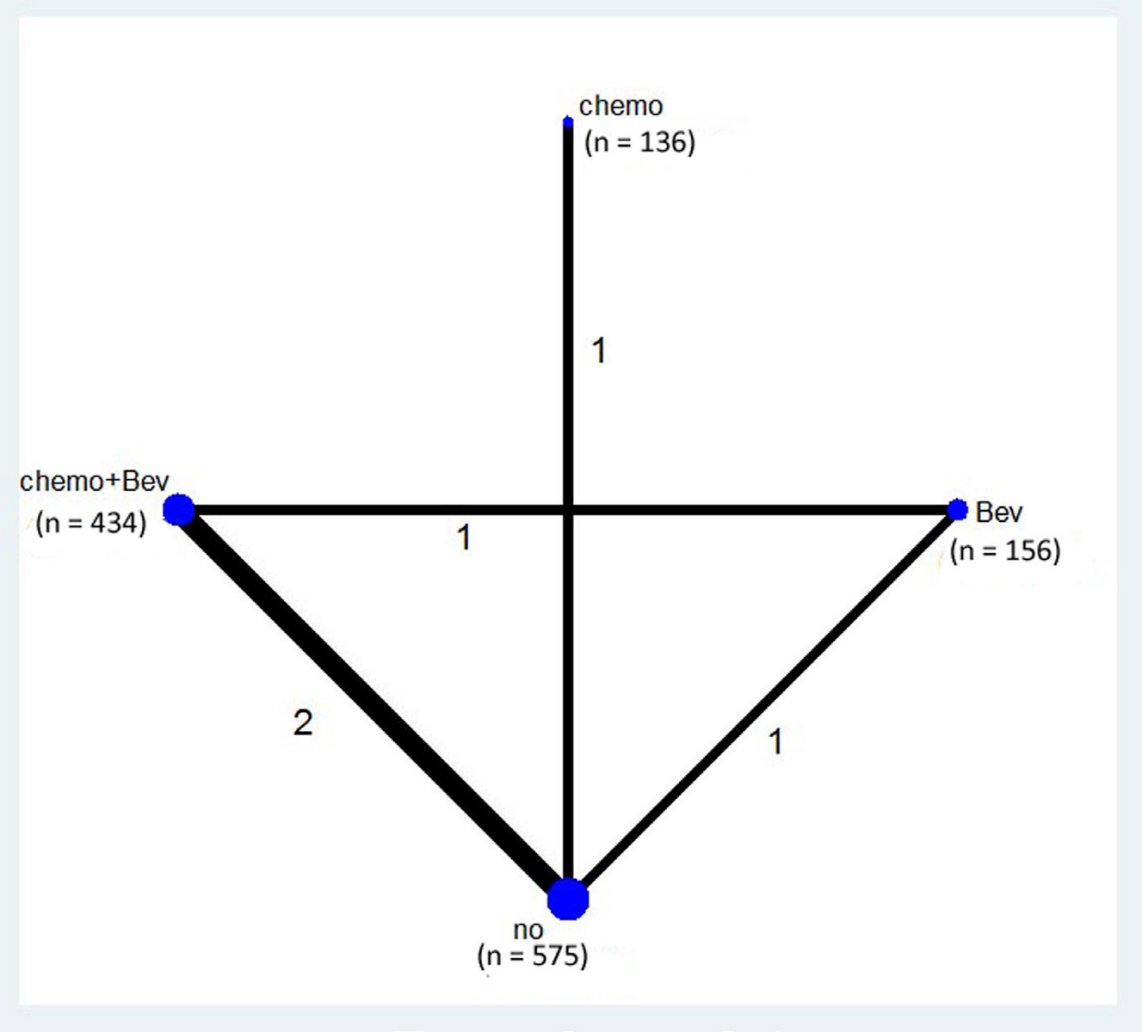
Network of the comparisons included in the network meta-analysis The sizes of the nodes are proportional to the numbers of patients (in parentheses) randomized to receive the treatment. The width of the lines is proportional to the number of trials (next to the line) comparing the connected treatments.

The network of the comparisons is shown in Figure 2. Four regimens from three trails were included in the network. Two studies compared chemotherapy plus bevacizumab (Bev) as maintenance treatment with observation. One study compared Bev alone as maintenance treatment with observation. One study compared capecitabine as maintenance treatment with observation. One study compared chemotherapy plus Bev with Bev alone.

### Progression-free survival

**Figure 3:**
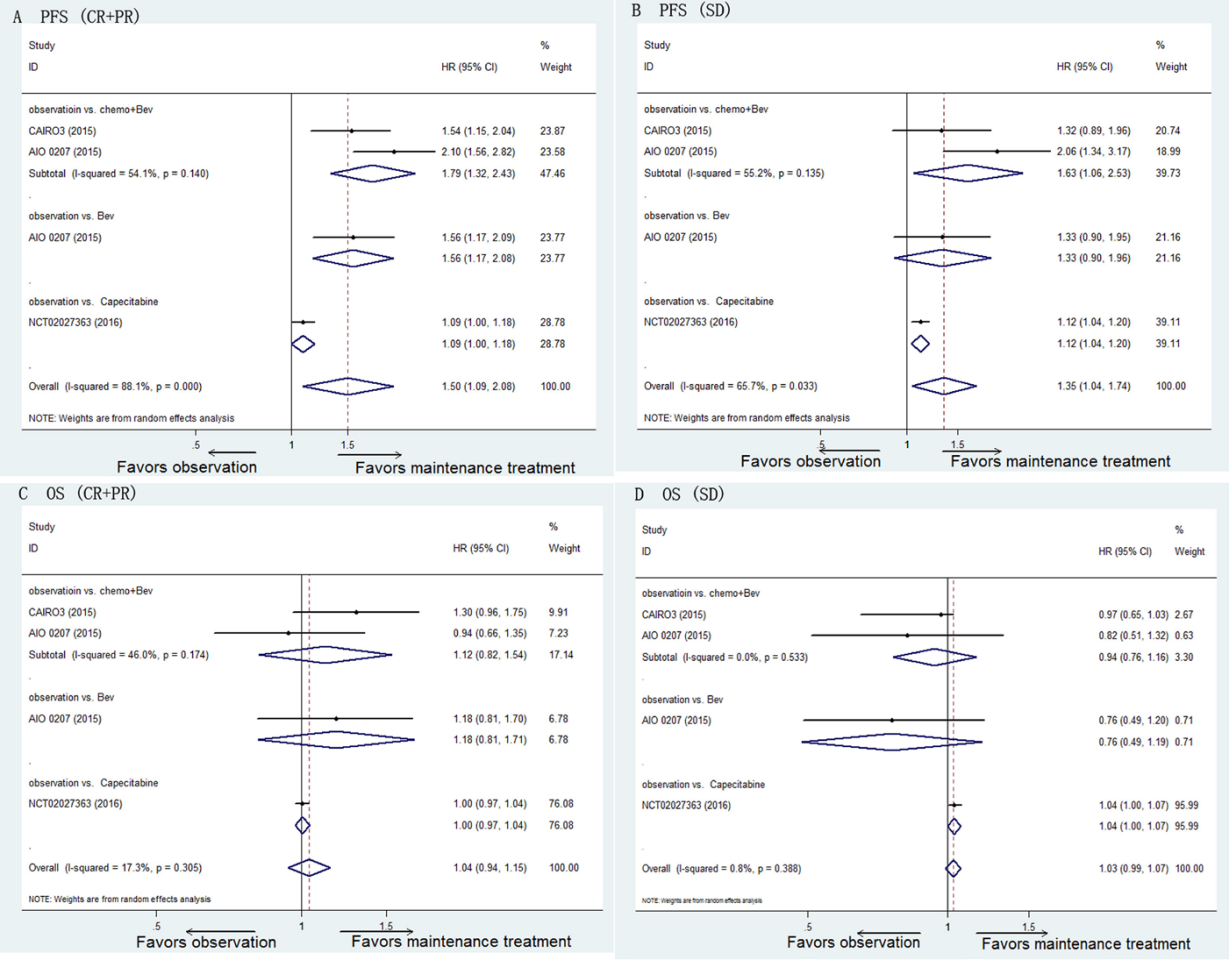
Pooled HRs of PFS for patients with CR/PR after induction therapy (**A**) and those with SD (**B**) and pooled HRs of OS for patients with CR/PR after induction therapy (**C**) and those with SD (**D**) determined using direct meta-analysis. Abbreviations: Bev, Bevacizumab; HR, hazard ratio; PFS, progression-free survival; OS, overall survival; CR, complete response; PR, partial response; SD, stable disease.

For the patients who achieved CR/PR after the induction treatment, maintenance treatment significantly prolonged the median PFS compared with observation (HR 1.50, 95% CI 1.09–2.08, *P* = 0.014). However, the heterogeneity was significant (I^2^ = 88.1%, *P* < 0.001). Chemotherapy plus Bev as maintenance treatment prolonged the median PFS compared with observation (HR 1.79, 95% CI 1.32–2.43, *P* < 0.001; I^2^ = 54.1%, *P* = 0.140) (Figure 3A).

Similarly, for the patients who achieved SD after the induction treatment, maintenance treatment significantly prolonged the median PFS compared with observation (HR 1.35, 95% CI 1.04–1.74, *P* = 0.024). However, the heterogeneity was significant (I^2^ = 65.7%, *P* = 0.033). Chemotherapy plus Bev as maintenance treatment prolonged the median PFS compared with observation (HR 1.63, 95% CI 1.06–2.53, *P* = 0.027; I^2^ = 55.2%, *P* = 0.135) (Figure 3B).

### Overall survival

For the patients who achieved CR/PR after the induction treatment, maintenance treatment prolonged the median OS compared with observation but the difference was not significant (HR 1.04, 95% CI 0.94–1.15, *P* = 0.477; I^2^ = 17.3%, *P* = 0.305) (Figure 3C).

For the patients who achieved SD after the induction treatment, maintenance treatment significantly prolonged the median OS compared with observation (HR 1.03, 95% CI 0.99–1.07, *P* = 0.121; I^2^ = 0.8%, *P* = 0.388) (Figure 3D).

### Network meta-analysis

Considering chemotherapy plus target therapy as the standard strategy of maintenance treatment, we set chemotherapy plus Bev as the basis of network meta-analysis.

**Figure 4:**
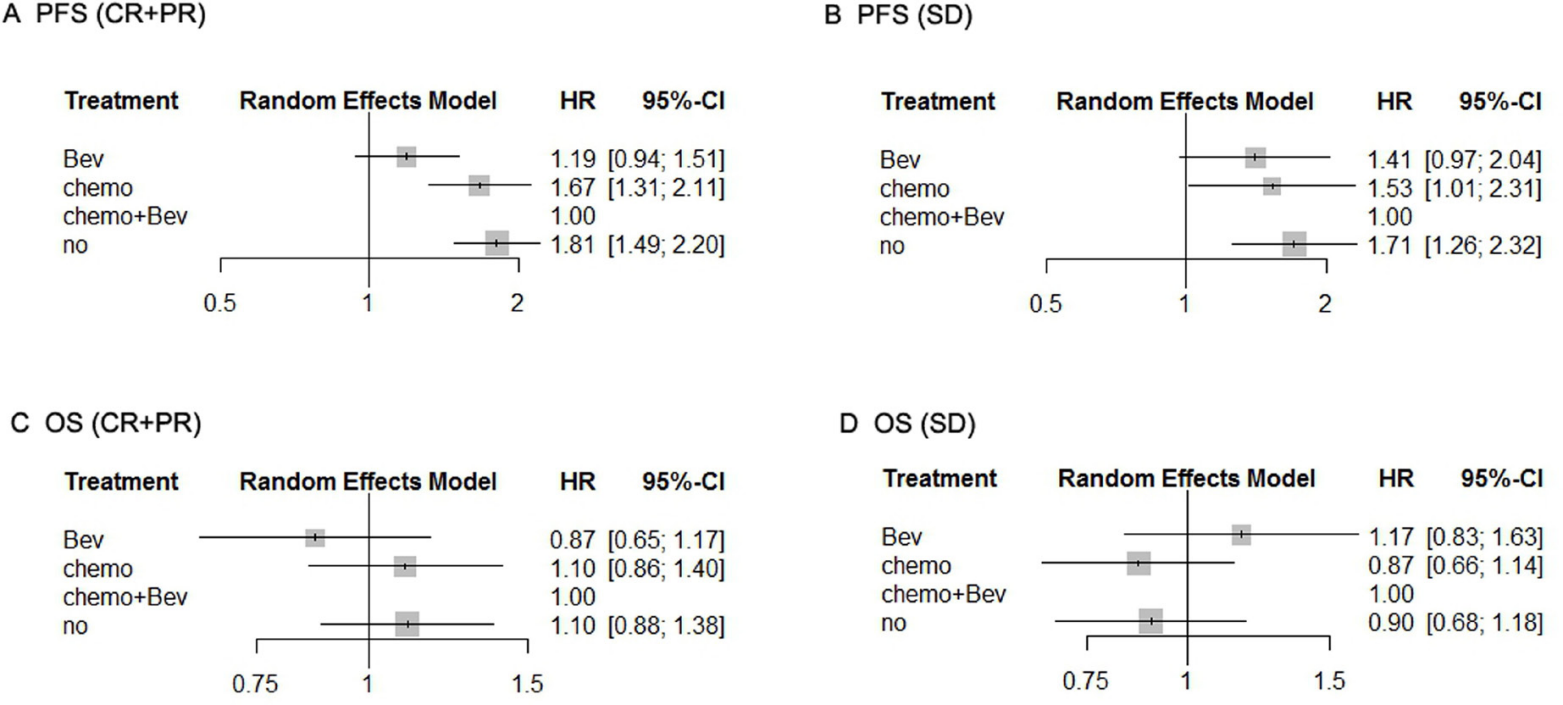
Pooled HRs of PFS for patients with CR/PR after induction therapy (**A**) and those with SD (**B**) and pooled HRs of OS for patients with CR/PR after induction therapy (**C**) and those with SD (**D**) determined using network meta-analysis. Abbreviations: Bev, Bevacizumab; HR, hazard ratio; PFS, progression-free survival; OS, overall survival; CR, complete response; PR, partial response; SD, stable disease.

Regarding PFS of the patients who achieved CR/PR after induction treatment, the efficacy of chemotherapy alone and observation were inferior compared with chemotherapy plus Bev (chemotherapy alone: HR 1.67, 95% CI 1.31–2.11; observation: HR 1.81, 95% CI 1.49–2.20). There was no difference between Bev alone and chemotherapy plus Bev (HR 1.19, 95% CI 0.94–1.51) (Figure 4A). For PFS of the patients who achieved SD after induction treatment, the efficacy of chemotherapy alone and observation were also inferior compared with chemotherapy plus Bev (chemotherapy alone: HR 1.53, 95% CI 1.01–2.31; observation: HR 1.71, 95% CI 1.26–2.32) and there was no difference between Bev alone and chemotherapy plus Bev (HR 1.41, 95% CI 0.97–2.04) (Figure 4B).

With respect to OS, for the patients who achieved CR/PR after induction treatment, there was no difference between the four regimens of maintenance treatment (chemotherapy alone: HR 1.10, 95% CI 0.86–1.40; observation: HR 1.10, 95% CI 0.88–1.38; Bev alone: HR 0.87, 95% CI 0.65–1.17) (Figure 4C). Similar results were obtained for OS of patients who achieved SD after induction treatment (chemotherapy alone: HR 0.87, 95% CI 0.66–1.14; observation: HR 0.90, 95% CI 0.68–1.18; Bev alone: HR 1.17, 95% CI 0.83–1.63) (Figure 4D).

## DISCUSSION

This meta-analysis suggested that the patients who achieved CR/PR after the induction treatment benefited more from maintenance treatment than those who showed SD (PFS: HR for CR/PR 1.50 vs. HR for SD 1.35; OS: HR for CR/PR 1.04 vs. HR for SD 1.03). The results of network meta-analysis suggested that chemotherapy alone and observation were inferior to chemotherapy plus Bev as maintenance treatment.

The evaluation of the significance of maintenance treatment without oxaliplatin after induction treatment remains a front issue because of the dose-related cumulative occurrence of neuropathy [11]. The published randomized controlled trials (RCTs) focused on the efficacy of maintenance treatment of all patients without progression after the induction treatment; however, the benefit of maintenance treatment was not evident, especially for OS. It is essential to consider the heterogeneity among the total patients. In particular, patients who achieve CR/PR after the induction treatment may respond differently to the maintenance treatment from patients with SD. Furthermore, there might be heterogeneity between the patients who achieve SD with a less than 20% increase in the sum of diameters of lesions and those with SD with a less than 30% decrease in the sum of diameters of lesions after induction treatment [3]. The patients who achieved SD after induction treatment with a less than 20% increase in the sum of diameters of lesions might be resistant to the induction treatment. Our meta-analysis involved the studies in which subgroup analyses of the patients CR/PR and SD after induction treatment were available (CAIRO3 study, AIO 0207 study and NCT02027363 study). But heterogeneity of design existed among these studies which was the limitation of this meta-analysis. CAIRO3 study investigated the efficacy of maintenance treatment with capecitabine plus Bev versus observation after an 18 weeks (6 cycles) of induction treatment with CAPOX-B (capecitabine 1000 mg/m^2^ orally twice daily on days 1 to 14, oxaliplatin 130 mg/m^2^ intravenously on day 1, and Bev 7.5 mg/kg intravenously on day 1). AIO 0207 study investigated the efficacy of maintenance treatment with fluoropyrimidine plus Bev versus Bev versus observation after a 24 weeks of induction treatment with CAPOX (capecitabine 1000 mg/m^2^ orally twice daily on days 1–14, 22–35; oxaliplatin 70 mg/m^2^ intravenously on day 1, 8, 22, 29; Bev 7.5 mg/kg intravenously on day 1, 22), XELOX (capecitabine 1000 mg/m^2^ orally twice daily on days 1–14, 22–35; oxaliplatin 130 mg/m^2^ intravenously on day 1, 22; Bev 7.5 mg/kg intravenously on day 1, 22), FOLFOX6 (5-FU 400 mg/m^2^ intravenously bolus, and 5-FU 2400 mg/m^2^ intravenously on days 1, 15, 29; oxaliplatin 100 mg/m^2^ intravenously on days 1, 15, 29; [dl-]Folinic acid 400 mg/m^2^ intravenously on days 1, 15, 29; Bev 5 mg/kg intravenously on days 1, 15, 29), FOLFOX4 (5-FU 400 mg/m^2^ intravenously bolus, and 5-FU 600 mg/m^2^ intravenously on days 1, 2, 15, 16, 29, 30; oxaliplatin 85 mg/m^2^ intravenously on days 1, 15, 29; [dl-]Folinic acid 200 mg/m^2^ intravenously on days 1, 2, 15, 16, 29, 30; Bev 5 mg/kg intravenously on days 1, 15, 29), modified FOLFOX7 (5-FU 3000 mg/m^2^ intravenously on days 1, 15, 29; oxaliplatin 100 mg/m^2^ intravenously on days 1, 15, 29; [dl-]Folinic acid 400 mg/m^2^ intravenously on days 1, 15, 29; Bev 5 mg/kg intravenously on days 1, 15, 29), modified FOLFOX4 (5-FU 3000 mg/m^2^ intravenously on days 1, 15, 29; oxaliplatin 85 mg/m^2^ intravenously on days 1, 15, 29; [dl-]Folinic acid 400 mg/m^2^ intravenously on days 1, 15, 29; Bev 5 mg/kg intravenously on days 1, 15, 29), or simplified FOLFOX4 (5-FU 400 mg/m^2^ intravenously bolus, and 5-FU 2400 mg/m^2^ intravenously on days 1, 15, 29; oxaliplatin 85 mg/m^2^ intravenously on days 1, 15, 29; [dl-]Folinic acid 400 mg/m^2^ intravenously on days 1, 15, 29; Bev 5 mg/kg intravenously on days 1, 15, 29). NCT 02027363 study investigated the efficacy of maintenance treatment with capecitabine versus observation after an 18–24 weeks of induction treatment with XELOX (capecitabine 1000 mg/m^2^ orally twice daily on days 1–14, 22–35; oxaliplatin 130 mg/m^2^ intravenously on day 1, 22; Bev 7.5 mg/kg intravenously on day 1, 22) or FOLFOX (5-FU 400 mg/m^2^ intravenously bolus, and 5-FU 2400 mg/m^2^ intravenously every 2 weeks; oxaliplatin 85 mg/m^2^ intravenously on days 1, every 2 weeks; leucovorin 400 mg/m^2^ intravenously on days 1, every 2 weeks). CAIRO3 study suggested that CR/PR patients after induction treatment benefited more from the maintenance treatment with capcitabine plus Bev than SD patients. But AIO 0207 study suggested that, for the efficacy of maintenance treatment, there was no significant difference between CR/PR patients and SD patients. The difference of efficacy might due to the different length of induction treatment and different dosage of oxaliplatin between two studies. The length of induction treatment was longer in AIO 0207 study (24 weeks) than CAIRO3 study (18 weeks). And the dosage of oxaliplatin in AIO 0207 study (1020–1200 mg/m^2^) was higher than CAIRO3 study (780 mg/m^2^). After exclusion the patients progressed or intolerant to toxicity during induction treatment, more patients in CAIRO3 study achieved reintroduction treatment compared with AIO 0207 study (maintenance group: CAIRO3: 47% vs. AIO 0207: 19%; observation group: CAIRO3: 60% vs. AIO 0207: 46%). Considering 128 patients progressed and 52 patients dropped out of the study due to the unacceptable toxicity after 24 weeks of induction treatment in AIO 0207 study, 18 weeks might be the better length of induction treatment rather than 24 weeks. And these 180 patients might be still stable disease after 18 weeks of induction treatment. This means some SD patients of AIO 0207 study were not the true SD patients to induction treatment but CR/PR patients after 18 weeks of induction treatment.

Goey K, et al. published a meta-analysis during 2016 ESMO, which included CAIRO3 and AIO 0207 study. They compared the efficacy of chemo plus Bev with observation. They showed the result of PFS but not OS. And they suggested there was no significant difference of PFS between chemo plus Bev and observation. However, our meta-analysis included three studies and performed PFS and OS. Our results suggested CR/PR patients achieved more benefit from chemo plus Bev than SD patients (OS of CR/PR patients: observation vs. chemo plus Bev: HR 1.12; OS of SD patients: observation vs. chemo plus Bev: HR 0.94). Furthermore, the results of our network meta-analysis showed that CR/PR patients benefited more from the addition of Bev as maintenance treatment, which recommended chemo plus Bev as maintenance treatment of mCRC patients.

This meta-analysis had certain strengths. First, we analyzed the efficacy of maintenance treatment in subgroups of patients with CR/PR and those with SD. Second, network meta-analysis provided an indirect comparison of four regimens as maintenance. However, there were also some limitations in this meta-analysis. First, there was heterogeneity of design among the included studies which was discussed above. Second, the number of papers with available data of subgroup analysis was too small. Further RCTs are needed to analyze the efficacy of maintenance treatment in subgroups of patients with CR/PR and those with SD, respectively. We also appeal to the sponsors of RCTs to share their data for subgroup analyses.

In conclusion, mCRC patients with CR/PR after induction therapy benefitted more from maintenance treatment than patients with SD. Chemotherapy plus Bev was the most appropriate regimen for maintenance treatment.

## MATERIALS AND METHODS

### Search strategy

This network meta-analysis was performed according to the Preferred Reporting Items for Systematic Reviews and Meta-Analyses (PRISMA) guidelines [14]. We searched the abstracts of RCTs to evaluate the efficacies of maintenance treatment of mCRC patients. The resources searched included PubMed, Cochrane Systematic Reviews, the Cochrane Collaboration Central Register of Controlled Clinical Trials, ClinicalTrials.gov, and the databases of the European Society for Medical Oncology and American Society of Clinical. The search included articles dated from the inception of these resources until June 20, 2017 (the list of search terms is included in the APPENDIX). We also reviewed the bibliographies of these reports and related reviews to identify additional articles. These papers were subjected to manual searches. Finally, only the studies had the subgroup analysis of CR/PR and SD patients after induction treatment were included in our meta-analysis (Figure 1).

### Quality assessment and data extraction

Two investigators (Z-MY, F-LY) independently reviewed the entire text of eligible studies. Information was extracted and inserted into an electronic database that included patient characteristics, inclusion and exclusion criteria, treatment protocols, and outcomes. Any disagreement between reviewers was discussed with the other coauthors until a consensus was reached.

### Data synthesis and analysis

Outcomes of this study included PFS and OS. Random-effects models were used to account for the heterogeneity among studies. Standard meta-analysis was performed using Stata 12.1 (StataCorp, College Station, TX, USA). Network meta-analysis was performed using a netmeta package developed according to the theories of a classical frequentist setting included in the R language framework.

## SUPPLEMENTARY MATERIALS


