# Supplementary material for: Who will benefit more from maintenance therapy of metastatic colorectal cancer?
Source: Oncotarget. 2017 Dec 21;9(15):12479–86. doi: 10.18632/oncotarget.23549 (PMC5844763; doi:10.18632/oncotarget.23549)
Supplement: Supplementary file 1 [file oncotarget-09-12479-s001.pdf]

Who will benefit more from maintenance therapy of metastatic colorectal cancer?

SUPPLEMENTARY MATERIALS

APPENDIX

Search strategy

Pubmed:

("metastatic colorectal cancer"[Title/Abstract] OR "Colorectal Neoplasms"[MeSH Terms] OR "Colonic Neoplasms"[MeSH Terms] OR "Sigmoid Neoplasms"[MeSH Terms] OR "Rectal Neoplasms"[MeSH Terms]) AND (Humans[MeSH Terms] OR Adult[MeSH Terms] OR Aged[MeSH Terms] OR "Middle Aged"[MeSH Terms] OR "Young Adult"[MeSH Terms] OR Male[MeSH Terms] OR Female[MeSH Terms]) AND ("Organoplatinum Compounds"[MeSH Terms] OR chemotherapy*[Title/Abstract] OR Fluorouracil[MeSH Terms] OR Capecitabine[Title/Abstract] OR Tegafur[Title/Abstract] OR Leucovorin[Title/Abstract] OR Irinotecan[Title/Abstract] OR "Antineoplastic Combined Chemotherapy Protocols"[MeSH Terms] OR "Angiogenesis Inhibitors"[MeSH Terms] "Receptor, Epidermal Growth Factor"[MeSH Terms] OR "Antibodies, Monoclonal"[MeSH Terms] OR "Antibodies, Monoclonal, Humanized"[MeSH Terms] OR "Antibodies, Monoclonal, Murine-Derived"[MeSH Terms] OR "Molecular Targeted Therapy"[MeSH Terms] OR bevacizumab[Title/Abstract] OR cetuximab[Title/Abstract] OR panitumumab[Title/Abstract] OR ramucirumab[Title/Abstract] OR matuzumab[Title/Abstract] OR aflibercept[Title/Abstract]) AND ("Follow-Up Studies"[MeSH Terms] OR "Treatment Outcome"[MeSH Terms] OR "Prognosis"[MeSH Terms] OR "Survival Rate"[MeSH Terms] "Survival Analysis"[MeSH Terms] OR "Quality of Life"[MeSH Terms]) AND ("Randomized Controlled Trial" [Publication Type] NOT ("Clinical Trial, Phase I"[Publication Type] OR "second-line"[Title/Abstract]))

Cochrane Library:

http://onlinelibrary.wiley.com/cochranelibrary/search/advanced?hiddenFields.strategySortBy=last-modified-date;desc&hiddenFields.showStrategies=false&hiddenFields.containerId=9122339467944699691&hiddenFields.originalContainerId=&hiddenFields.etag=7574179336855920768&meshOrBasicAppended=true#

American Society of Clinical Oncology database of abstracts:

Metastatic colorectal cancer (http://meetinglibrary.asco.org/abstracts)

ClinicalTrials.gov:

Category: "Carcinoma, Colorectal" (http://clinicaltrials.gov/)
